# Dual-Role of Cholesterol‐25‐Hydroxylase in Regulating Hepatitis B Virus Infection and Replication

**DOI:** 10.1128/mbio.00677-22

**Published:** 2022-05-19

**Authors:** Qi Wei, Hongxiao Song, Yanli Gao, Fengchao Xu, Qingfei Xiao, Fei Wang, Bingxin Lei, Junqi Niu, Pujun Gao, Haichun Ma, Guangyun Tan

**Affiliations:** a Center for Pathogen Biology and Infectious Diseases, Department of Immunology, Key Laboratory of Organ Regeneration and Transplantation of the Ministry of Education, the First Hospital of Jilin University, Changchun, Jilin, China; b Department of Anesthesiology, the First Hospital of Jilin University, Changchun, Jilin, China; c Department of Pediatrics, the First Hospital of Jilin University, Changchun, Jilin, China; d Department of Nephrology, the First Hospital of Jilin University, Changchun, Jilin, China; e Department of Hepatology, The First Hospital of Jilin University, Changchun, Jilin, China; Duke University Medical Center

**Keywords:** CH25H, 25HC, HBV, HBx, hepatitis B virus

## Abstract

Hepatitis B virus (HBV)‐related diseases are among the major diseases that affect millions of people worldwide. These diseases are difficult to eradicate and thus pose a serious global health challenge. There is an urgent need to understand the cross talk mechanism between HBV and the host. Cholesterol‐25‐hydroxylase (CH25H) and its enzymatic product, 25‐hydroxycholesterol (25HC), were previously shown to exhibit effective broad‐spectrum antiviral activity. However, the role of CH25H in the regulation of HBV infection and replication remains unclear. The present study reported increased expression of CH25H in HBV-infected patients compared to healthy subjects. Importantly, higher expression of CH25H expression was found to be associated with low HBV replication. Additionally, the present study aimed to identify CH25H mutants, which would lack hydroxylase activity but retain antiviral activity toward HBV infection and replication. Interestingly, it was observed that both CH25H and its mutants interacted with HBx protein and inhibited nuclear translocation of HBx. In particular, CH25H interacted with the C-terminal region of HBx, while transmembrane region 3 of CH25H was found to be critical for CH25H–HBx interaction and inhibition of HBV replication. The study results suggested that 25HC promoted HBV infection but not HBV replication. Thus, the results of the present study suggested the involvement of a dual mechanism in CH25H-mediated regulation of HBV replication. The study clearly demonstrated cross talk between HBV and the host through CH25H–HBx axis.

## INTRODUCTION

In the past few decades, a continuous outbreak of highly contagious viruses has emerged as a serious global health challenge. Hundreds millions of people are still suffering from coronavirus disease 2019 (COVID-19) caused by SARS-CoV-2 (severe acute respiratory syndrome coronavirus 2) (https://covid19.who.int/). Therefore, there is an urgent need to develop novel strategies to control viral infectious diseases. Cholesterol‐25‐hydroxylase (CH25H) is a member of the redox enzyme family, and it has 272 amino acids (aa). It is also known as cholesterol‐25‐monooxygenase. In human cells, it is characterized by the presence of a transmembrane domain and FA domain. The endoplasmic reticulum (ER) and Golgi apparatus are the main cellular locations of CH25H, wherein CH25H catalyzes the oxidation of cholesterol to 25HC (1). CH25H is known to play multiple biological roles, especially in lipid metabolism, antiviral processes, inflammatory response, cell survival, and others (2). The enzymatic product of CH25H, 25‐hydroxycholesterol (25HC), has been previously shown to play a critical role in the blockage of cell–virus fusion in response to viral infection. Interestingly, 25HC is known to exhibit effective broad‐spectrum antiviral activity. Most of the viral infection processes have been shown to be stopped by 25HC treatment at the first stage. In particular, the antiviral activity of 25HC was previously established against a variety of viruses, including vesicular stomatitis virus, herpes simplex virus, murine hepatitis virus 68, ebola virus, Rift Valley fever virus, tick-borne encephalitis virus strain RSSE, hepatitis C virus (HCV), and Nipah viruses (3, 4). In addition to these, highly contagious viruses, like Zika (5) and SARS‐CoV‐2 ([Bibr B6][Bibr B7][Bibr B8]), were also efficiently inhibited by the action of 25HC. Oxygenated derivatives of cholesterol have been reported to enhance hepatitis B virus replication by activating the nuclear receptor LXRα (9), and we detected the action of CH25H in the process of host and virus interaction through monocytes (10). However, to the best of our knowledge, no study has yet explored the detailed mechanism of CH25H-mediated regulation of HBV.

In the host, interferon, especially type I interferon production, acts as the first line of defense against viral infection. The induction of interferon leads to the upregulation of hundreds of interferon-stimulated genes (ISGs), which include *CH25H* (11). Previous studies conducted in our laboratory screened 145 ISGs, 26 of which interacted with HBx protein, a small positive regulatory factor of HBV, and several ISGs were found to inhibit HBV replication. These included *TRIM5γ*, *Apobec3G*, *CBFβ*, *TRIM14*, and *TRIM25* (12–17). In general, HBV is a partially double‐stranded DNA virus. HBV viral RNAs are usually transcribed from covalently closed circular DNA (cccDNA) that resides in the nuclei of infected hepatocytes (18). HBx was suggested to benefit HBV replication by promoting proteasome‐mediated degradation of structural maintenance of chromosomes 5/6 (Smc5/6), which maintains the stability of chromosomes and suppresses the gene expression from episomal DNA, such as HBV cccDNA (19, 20). Interestingly, the degradation of Smc5/6 was reported to play a critical role in the accumulation of DNA damage, which might result in HBx-mediated tumorigenesis (21). It was previously shown that HBx does not directly bind to the DNA. However, HBx always acts as a transactivator of viral replication, which is mediated via the recruitment of other factors to the cccDNA minichromosome (22). Similarly, HBx has been shown to play an important role in the regulation of host gene expression (23).

The present study aimed to identify CH25H mutants that lacked hydroxylase activity but still exhibited antiviral activity and restricted HBV replication. The study showed that CH25H interacted with HBx protein. Importantly, this interaction significantly reduced the nuclear translocation of HBx, which resulted in the inhibition of HBx‐mediated transactivation of the cccDNA template. Additionally, 25HC was found to promote HBV infection in the HepG2‐NTCP and the PHH infection models. Altogether, the findings of the present study demonstrated a novel dual role of CH25H in the inhibition of HBV replication by acting on HBx and promotion of HBV infection by the production of 25HC. Further, CH25H–HB*x* axis might provide more insights into HBV–host interaction.

## RESULTS

### CH25H was upregulated in HBV patients, and it correlated with HBV replication.

**FIG 1:**
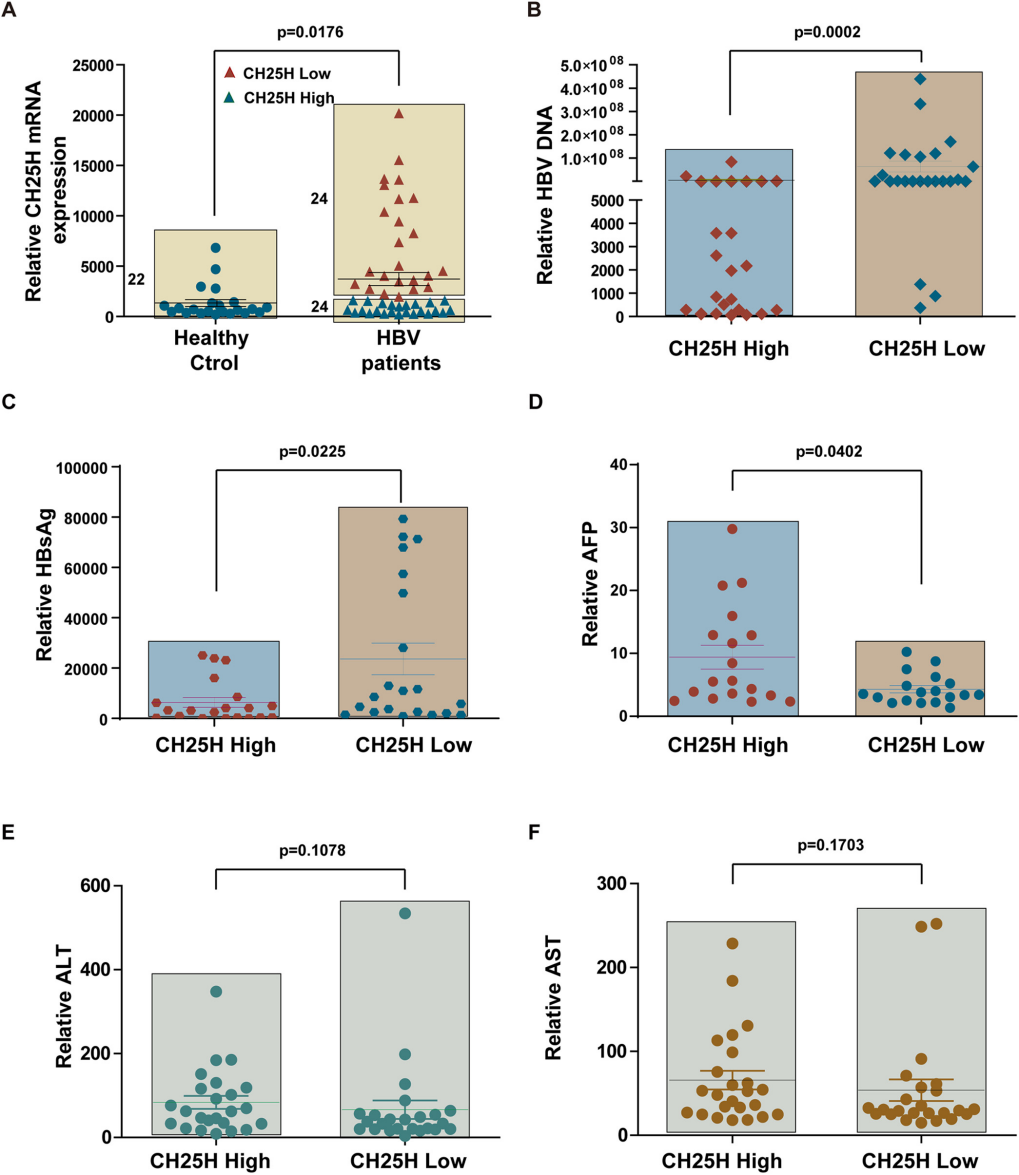
CH25H correlates with HBV replication in HBV patients. (A) PBMCs from healthy control (Ctrol; 22) and HBV patients (48) were isolated and subjected to RNA extraction; qPCR was performed to evaluate the CH25H mRNA levels. (B to F) HBV patients were categorized into two groups according to the expression of CH25H: as CH25H high and low groups. The medical information of the patients was collected from the First Hospital of Jilin University. (B) HBV DNA copies and (C) HBsAg, (D) AFP, (E) ALT, and (F) AST levels were analyzed according to the CH25H level. Mean (± standard deviation [SD]) values from three independent experiments are shown. *P* < 0.05 was considered to indicate statistical significance.

CH25H was previously reported to be induced in HCV patients (24). Type I interferon (IFN) has been shown to be induced in HCV- but not in HBV-infected patients. The present study first aimed to investigate the expression of CH25H in HBV patients. In particular, samples were collected from healthy control subjects and HBV patients, and quantitative real-time PCR (qRT‐PCR) was performed to determine mRNA levels of CH25H. Interestingly, CH25H was found to be significantly upregulated in the patient samples (Fig. 1A). Next, we assessed whether the expression of CH25H was associated with HBV replication. In particular, the patients were divided into two groups, namely, the high- and low-CH25H groups. As expected, HBV DNA copies were found to be significantly lower in the high-CH25H group (Fig. 1B). Similar results were recorded for the serum HBsAg levels and AFP levels (Fig. 1C and D). The study also assessed and compared aspartate transaminase (AST) and alanine aminotransferase (ALT) levels in the two groups. However, no significant differences were recorded (Fig. 1E and F), which indicated that CH25H expression correlated with HBV replication but not with liver function. Thus, all these results indicated a higher expression of CH25H in HBV patients, wherein the expression of CH25H was negatively correlated with HBV replication.

### CH25H-mediated inhibition of HBV replication and infection were independent of its enzyme activity.

**FIG 2:**
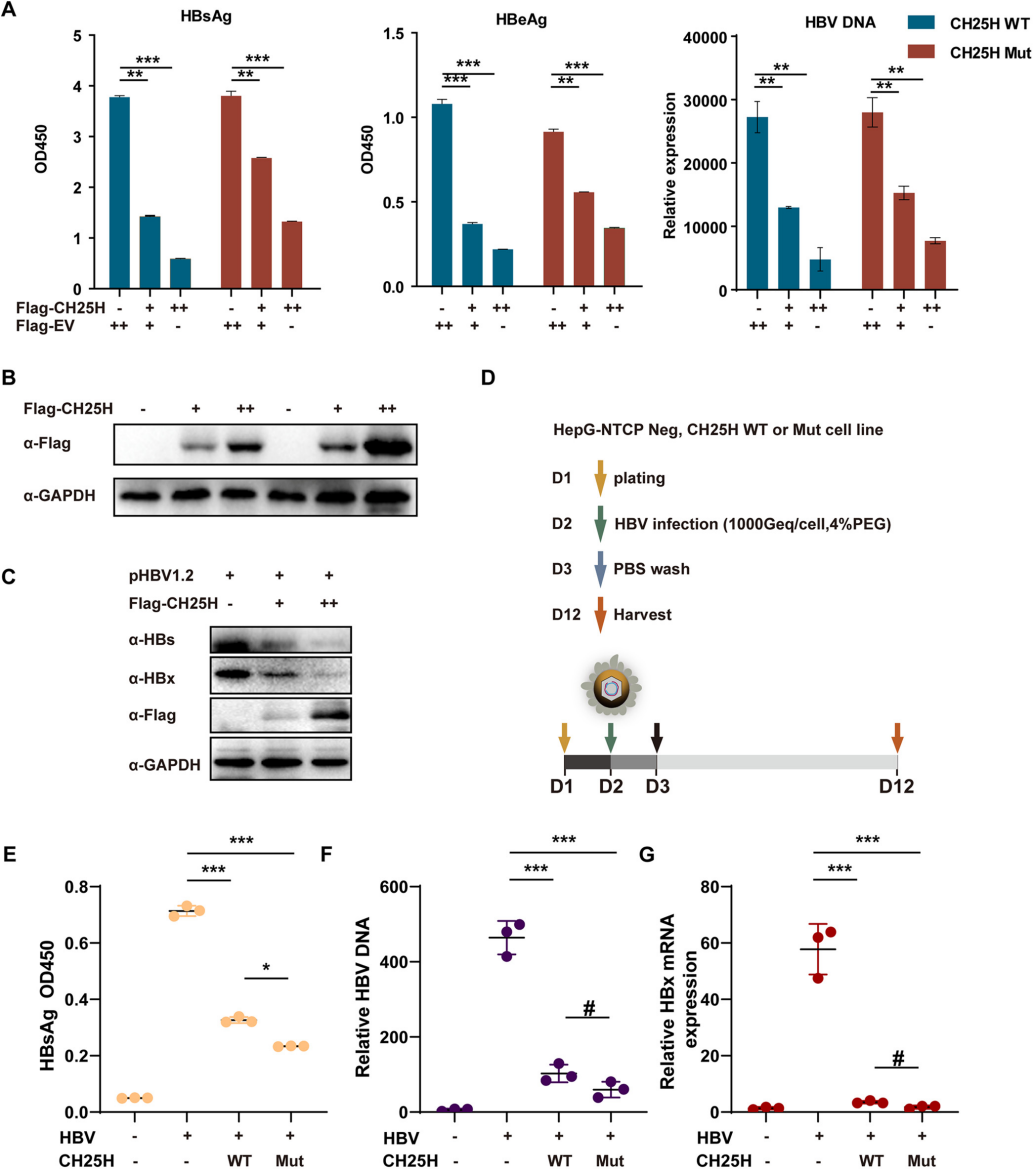
CH25H but not 25HC inhibits HBV replication. (A) HepG2 cells were transfected with pHBV1.2 or cotransfected with Flag-tagged CH25H expression plasmids as indicated. 48 h later, and the cells and supernatant were harvested and subjected to qPCR or ELISA. OD450, optical density at 450 nm. (B) Lysate of cells from panel A was subjected to immunoblotting with the Flag or GAPDH antibody, as indicated. (C) HepG2 cells were transfected with Flag-CH25H or pHBV1.2 plasmid as indicated; after 48 h, the cells were harvested and the whole-cell lysates were subjected to immunoblotting using anti-Flag, anti-HBx, anti-HBs, or anti-GAPDH antibodies. (D) The scheme of HBV infection. (E to G) HepG2-NTCP Neg, CH25H wild-type (WT), or Mut cells were plated in a 12-well plate (0.8 × 10^5^/well) infected with HBV for 24 h, followed by washing of the cells with PBS. After infection, the cells were washed with PBS and maintained for another 9 days, and the medium was changed every 2 days. The supernatant and cells were collected for the detection of HBV DNA, HBsAg, and HBx by qPCR or ELISA. Mean (±SD) values from 3 independent experiments are shown. **, *P* < 0.01; ***, *P* < 0.001.

Next, the study aimed to confirm the function of CH25H in HBV replication. It has been previously shown that H242G/H243G mutation in the human *CH25H* gene blocked the ability of CH25H to produce 25HC (4). Thus, in the present study, plasmids expressing wild-type (WT) and mutant (Mut) CH25H were cotransfected into HepG2 cells. As expected, after 48 h of transfection, the levels of pgRNA in the cells and HBsAg and HBeAg in the supernatant were found to be reduced in a dose-dependent manner (Fig. 2A and B; see also Fig. S1A in the supplemental material). In addition to this, HBx and HBs in the cells also showed similar inhibition in CH25H-transfected HepG2 cells (Fig. 2C). All these findings indicated a similarity in the ability of CH25H and the enzyme-dead mutants to inhibit HBV replication, which suggested that CH25H enzymatic activity was not needed for the regulation of the HBV replication process. Accordingly, HBV infection assay was performed to confirm this result. For this purpose, HepG2-NTCP cell line expressing CH25H WT or Mut was infected with HBV for 24 h; this infection assay indicated that both CH25H WT and Mut played a positive role in the HBV infection and replication process (Fig. 2D to G). The CH25H expression was analyzed by qRT-PCR (Fig. S1B). Altogether, these findings demonstrated that CH25H-mediated inhibition of HBV infection and replication was not dependent on enzyme activity.

10.1128/mbio.00677-22.1 FIG S1 (A) Cell viability after transfection (as shown in Fig. 2A) was analyzed. (B) HepG2-NTCP cells were infected with the Lentivirus neg, CH25H WT, or Mut, and after 48 h, the cells were selected by puromycin (2 μg/mL) until all cells showed GFP expression, followed by collection of the cells for the detection of CH25H. Mean (±SD) values from three independent experiments are shown. ***, *P* < 0.001; #, *P* > 0.05. Download FIG S1, TIF file, 0.5 MB. Copyright © 2022 Wei et al. 2022 Wei et al. https://creativecommons.org/licenses/by/4.0/ This content is distributed under the terms of the Creative Commons Attribution 4.0 International license.

### HBx was important for CH25H‐mediated inhibition of HBV replication.

**FIG 3:**
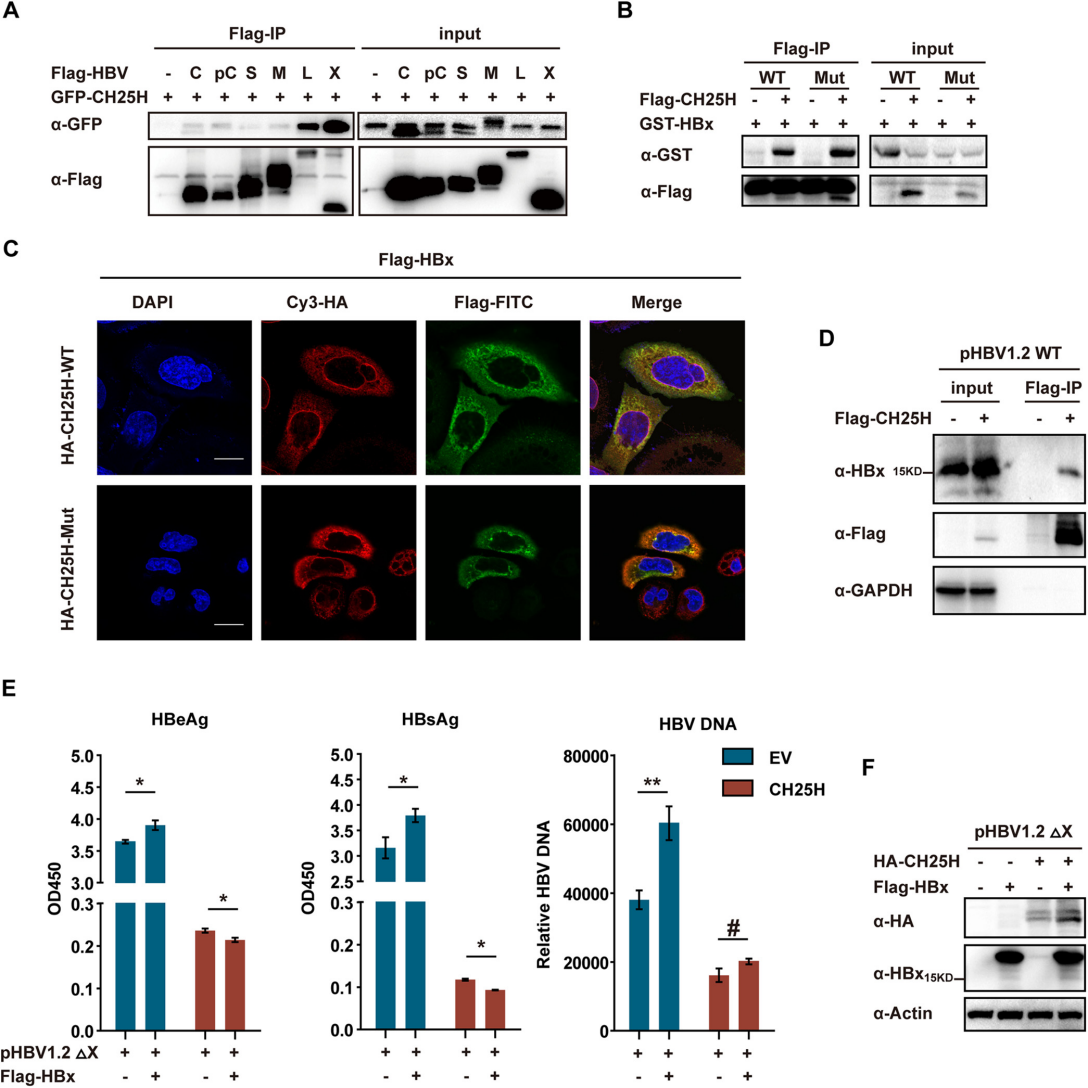
HBx is important in CH25H-mediated inhibition of HBV replication. (A) 293T cells were cotransfected with constructs expressing Flag-tagged HBV proteins (C, core; pC, preCore; S, small S; M, middle S; L, large S; X, HBx) and GFP-CH25H, as indicated. After 28 h, co-IP was performed and the cell lysates and precipitated samples were analyzed by immunoblotting using an anti-Flag or anti-GFP antibody. (B) HepG2 cells were cotransfected with constructs expressing Flag-CH25H and GST-HBx, as indicated; after 28 h, the cells were collected and analyzed as described for panel A. (C) HepG2 cells were transfected with Flag-HBx or cotransfected with HA-CH25H WT or Mut; after 36 h, the cells were washed with PBS and subjected to immunofluorescence with anti-HA or anti-Flag antibody. (D) HepG2 cells were transfected with pHBV1.2 or cotransfected with CH25H as indicated; after 48 h, the cells were collected and subjected to co-IP assay with an anti-Flag, anti-HBx, or anti-GAPDH antibody. (E) pHBV1.2 ΔX plasmids were transfected with Flag-HBx or HA-CH25H plasmids, as indicated; after 48 h, the cells and supernatant were harvested and subjected to qPCR or ELISA. (F) Lysate of cells from panel E was subjected to immunoblotting with anti-HBx, anti-HA, or anti-Actin antibodies, as indicated. Mean (±SD) values from three independent experiments are shown. *, *P* < 0.05; **, *P* < 0.01; #, *P* > 0.05.

Protein–protein interaction is known to play an indispensable role in the cross talk of the host and virus. Here, the interaction between CH25H and HBV proteins was assessed by coimmunoprecipitation (co‐IP) assay in 293T cells. A strong interaction was observed between CH25H and HBx protein (Fig. 3A). In addition to this, HBx also interacted with the mutant CH25H (Fig. 3B). The colocalization of HBx and CH25H (WT and Mut) was verified by immunofluorescence (Fig. 3C). The interaction of CH25H and HBx was further confirmed by expressing CH25H in pHBV-transfected HepG2 cells (Fig. 3D). Next, we assessed whether CH25H–HBx dimer played any role in HBV replication. To address this question, a pHBV1.2 plasmid without HBx expression was used (pHBV1.2ΔX). In pHBV1.2ΔX-transfected cells, HBV replication was significantly enhanced by HBx expression plasmids transfection. However, in the case of CH25H-cotransfected cells, HBeAg and HBsAg levels were found to be uniformly decreased in the supernatant of HBx-cotransfected cells and HBV DNA level was not significant, which suggested that the function of HBx in the promotion of HBV replication was blocked by CH25H (Fig. 3E and F). Altogether, these results clearly indicated that HBx was important for CH25H‐mediated inhibition of HBV replication.

### CH25H inhibited nuclear translocation of HBx protein.

**FIG 4:**
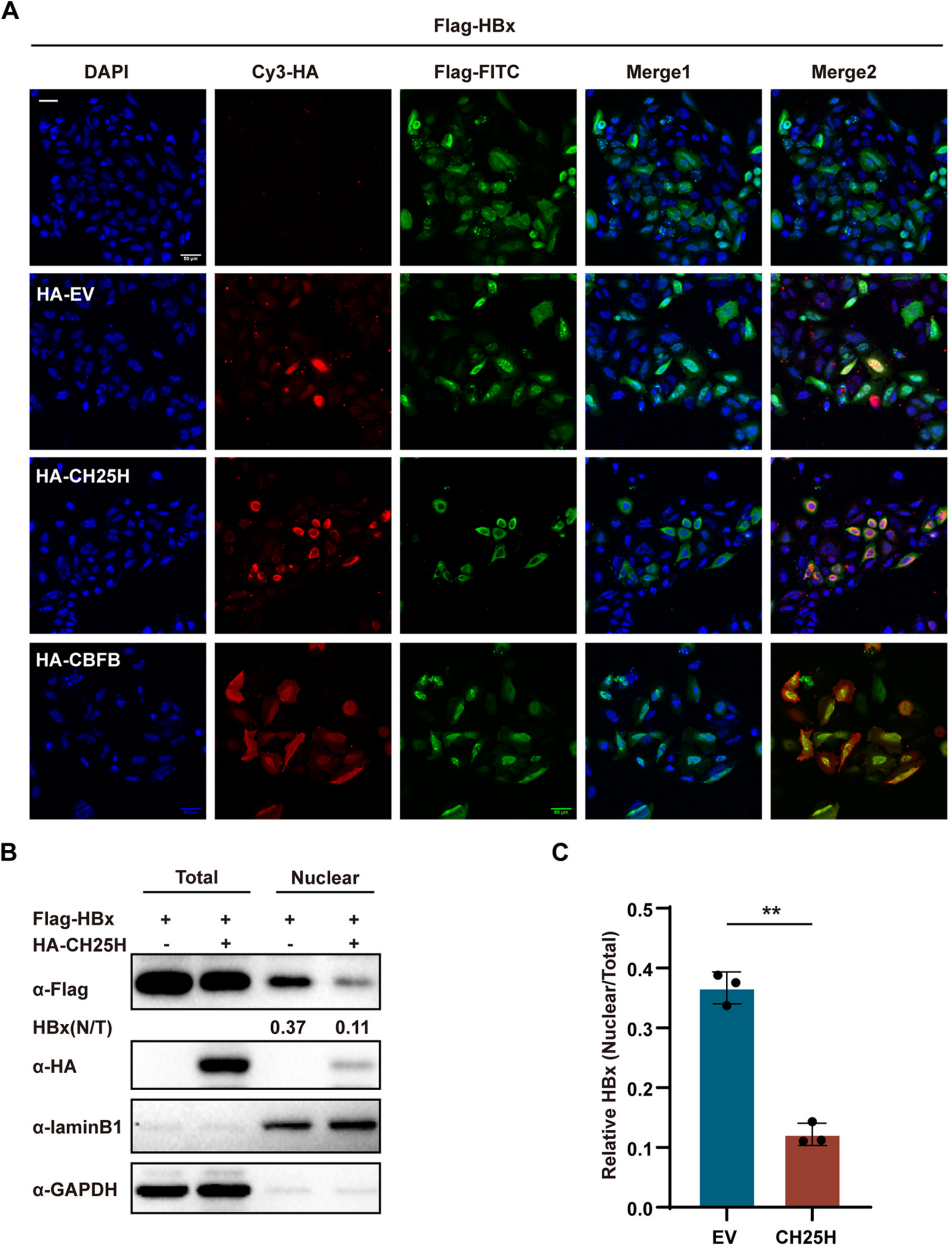
CH25H inhibits HBx nucleus translocation. (A) HepG2 cells were Flag-HBx transfected alone or cotransfected with constructs expressing HA-empty vector, CH25H, or CBFB, as indicated. After 36 h, the cells were washed with PBS and subjected to immunofluorescence with anti-HA or anti-Flag antibodies. (B and C) HepG2 cells were transfected with Flag-HBx or CH25H expression plasmids, as indicated; after 36 h, the cells were collected and the nucleus and cytoplasm proteins were isolated, and the lysate was then subjected to immunoblotting using Flag or HA antibodies, as indicated. Data are representative of at least three independent experiments.

Under the synergy of DDB1, HBx was previously reported to promote the degradation of Smc5/6, which maintains the stability of cccDNA (19). It was previously reported that TRIM14 and CBFβ play a role in the inhibition of the formation of the HBx‐DDB1‐Smc complex (13, 16). Next, the present study investigated whether CH25H–HBx interaction affected HBx‐mediated Smc5/6 degradation. To verify the interaction of HBx‐DDB1‐Smc in the presence of CH25H, a co-IP assay was performed. Importantly, no inhibition was observed in the cells overexpressing CH25H compared to the cells transfected with an empty vector, which indicated a negative role of CH25H in HBx‐DDB1‐Smc complex formation (Fig. S2). Nuclear translocation of HBx is necessary for the regulation of gene expression in viruses or hosts (25–27). Thus, we investigated whether CH25H–HBx interaction inhibited HBx transport. Interestingly, in the cells coexpressing CH25H, it was observed that most of the HBx protein existed in the cytoplasm. Comparatively, in the case of HBx transfection alone, empty vector cotransfection, or CBFB expression plasmids cotransfection, even more HBx protein was observed in the nucleus (Fig. 4A). This result was consistent with the data shown in Fig. 3C. To further confirm these results, Western blot analysis was performed to detect the expression of HBx protein in the cytoplasm and nucleus in the presence of CH25H. As expected, nuclear HBx expression was significantly inhibited in the cells overexpressing CH25H (Fig. 4B and C). Altogether, these results suggested that CH25H inhibits nuclear translocation of HBx and further inhibits the transcription of HBV genes.

10.1128/mbio.00677-22.2 FIG S2 CH25H plays a negative role in HBx-DDB1-Smc complex formation. 293T cells were cotransfected with *Flag-HBx* and HA-CH25H, as indicated; after 24 h, the cells were treated with MG132 (10 μM) for 6 h, and the cells were collected and subjected to co-IP, followed by the analyses of the cell lysates and the precipitated samples by immunoblotting using an anti-Flag, anti-HA, anti-DDB1, anti-Smc5/6, or anti-GAPDH antibody, as indicated. Download FIG S2, TIF file, 0.9 MB. Copyright © 2022 Wei et al. 2022 Wei et al. https://creativecommons.org/licenses/by/4.0/ This content is distributed under the terms of the Creative Commons Attribution 4.0 International license.

### CH25H interacted with the C terminus of HBx protein.

**FIG 5:**
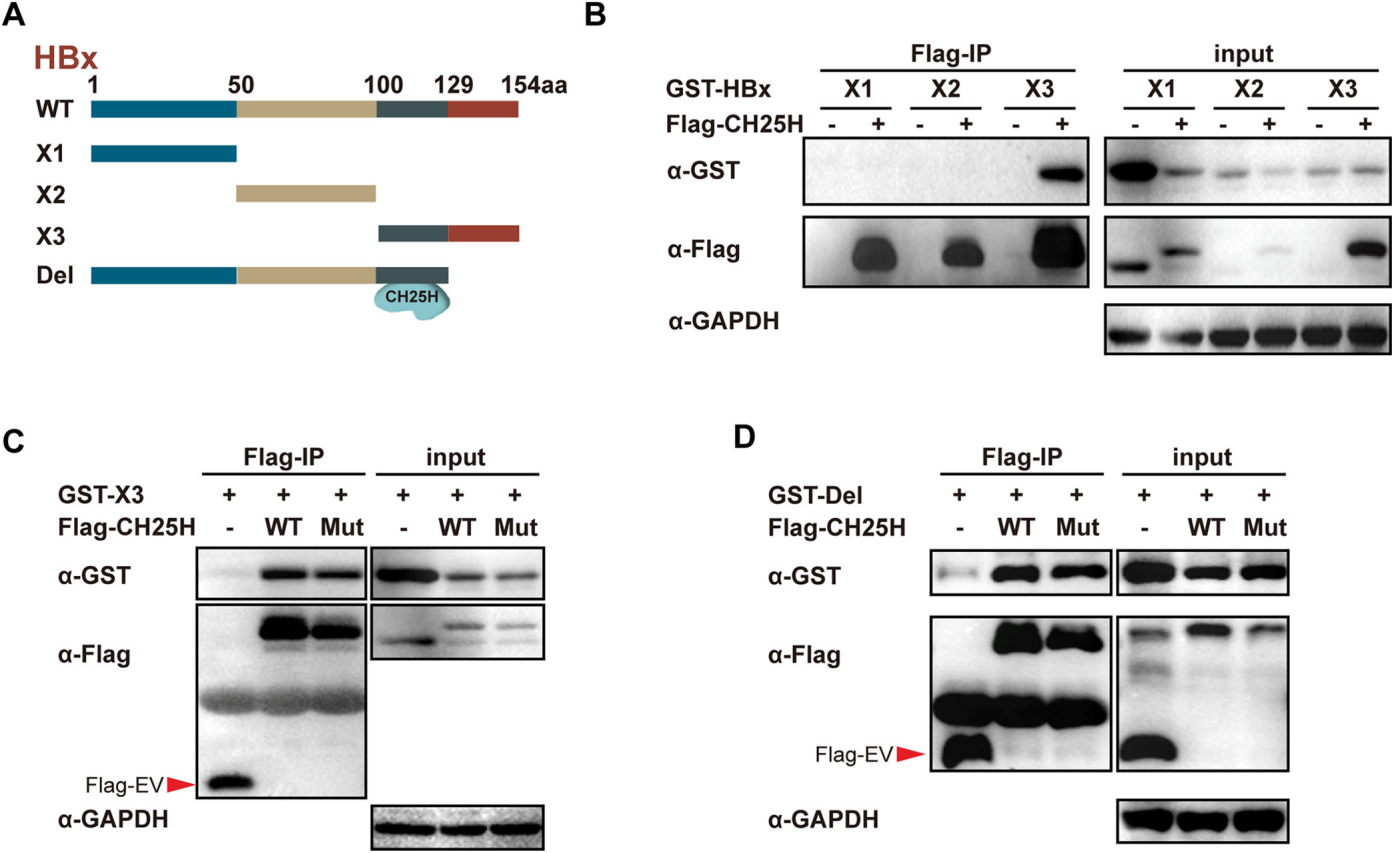
CH25H interacts with the C terminus of HBx protein. (A) Illustration of mutant HBx constructs, with the numbers indicating the amino acids in the HBx constructs. (B) 293T cells were cotransfected with Flag-CH25H and GST-HBx truncations or EV, as indicated; after 28 h, co-IP was performed and the cell lysates and precipitated samples were analyzed by immunoblotting using an anti-Flag, anti-GST, or anti-GAPDH antibody. (C) 293T cells were transfected with Flag-CH25H WT or Mut together with GST-HBx truncation 3, as indicated, and co-IP was performed and analyzed as described for panel B. (D) 293T cells were transfected with GST-HBx Del, Flag-CH25H WT, or Mut, as indicated, and analyzed as described for panel B. Data are representative of at least three independent experiments.

It was previously reported that the N terminus of HBx acts as a negative domain, and the functional regions are majorly focused on the C terminus (28, 29). Next, to identify the HBx region that interacted with CH25H, HBx was truncated into three parts, namely, X1, X2, and X3 (Fig. 5A). After cotransfection with Flag‐CH25H or Flag‐empty vector, a co-IP assay was performed. The results indicated that X3, but not X1 or X2, is bound to CH25H (Fig. 5B). In addition to this, we further confirmed that CH25H Mut protein, which lacked hydroxylase activity, was bound to X3 (Fig. 5C). Finally, a plasmid expressing HBx amino acids 1 to 129 (Del) (Fig. 5A) was used to verify binding regions more accurately. Interestingly, unlike TRIM5γ (which binds to aa 129 to 155), CH25H is bound to 100- to 129-amino-acid region, similar to TRIM14 (13, 14). Altogether, these results suggested that CH25H interacted with the C terminus of HBx.

### The transmembrane domain of CH25H was important for the interaction with HBx and inhibition of HBV replication.

**FIG 6:**
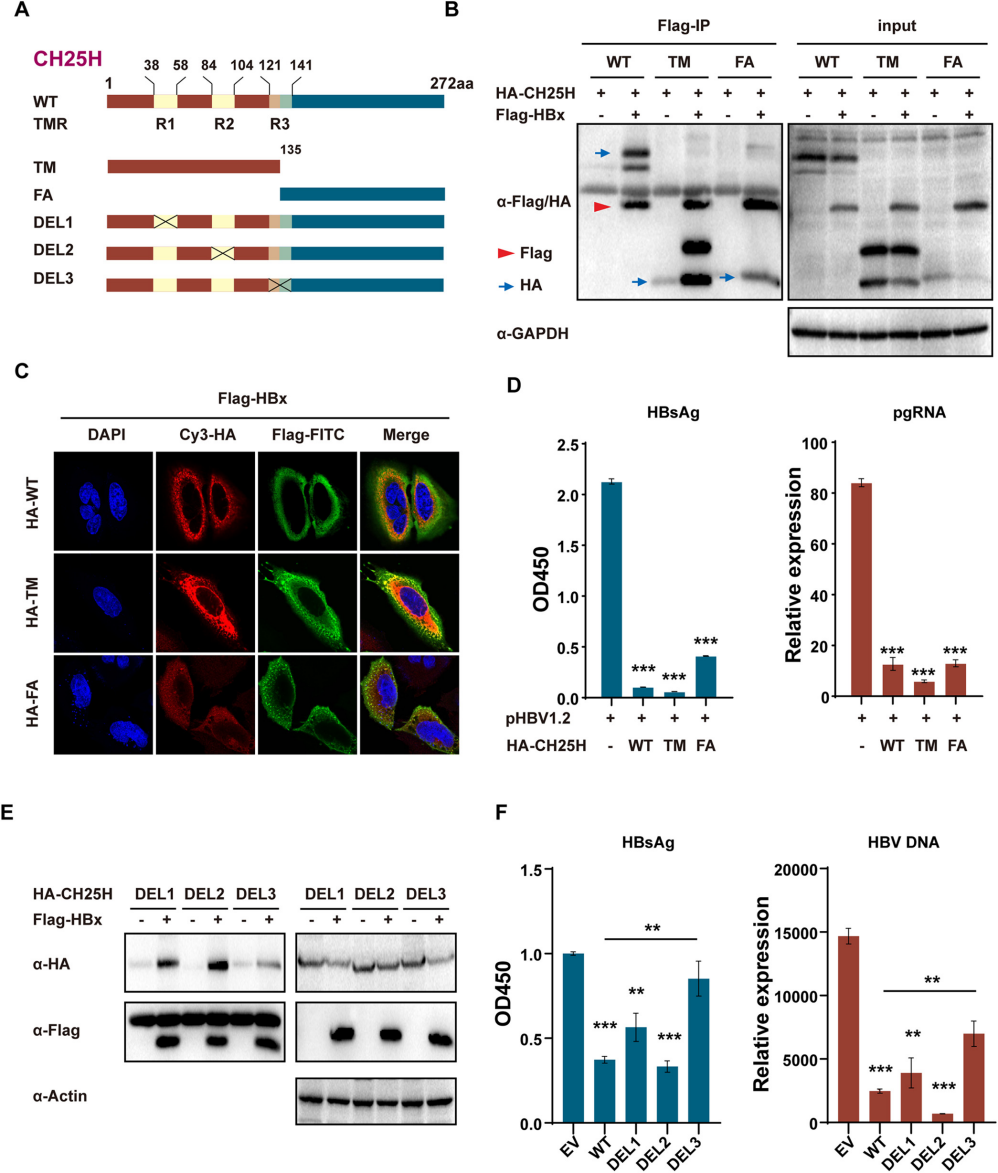
Ch25H transmembrane region 3 is important in CH25H-HBx interaction and the inhibition of HBV replication. (A) Illustration of mutant CH25H constructs, with the numbers indicating the amino acids in the CH25H constructs. (B) 293T cells were cotransfected with Flag-HBx and HA-CH25H WT or truncations, as indicated; after 28 h, co-IP was performed, and the cell lysates and precipitated samples were analyzed by immunoblotting using anti-Flag, anti-HA, or anti-GAPDH antibody. (C) HepG2 cells were transfected with Flag-HBx or cotransfected with HA-CH25H WT, Trans, or FA truncations; after 36 h, the cells were washed with PBS and then subjected to immunofluorescence with anti-HA or anti-Flag antibody. (D) HA-CH25H WT, Trans, or FA truncations were cotransfected with pHBV1.2 plasmids, as indicated; after 48 h, the cells and supernatant were harvested and subjected to qPCR or ELISA. (E) 293T cells were cotransfected with Flag-HBx and HA-CH25H Del1, Del2, or Del3, as indicated; after 28 h, co-IP was performed and analyzed as described for panel B. (F) HA-CH25H WT, Del1, Del2, or Del3 was cotransfected with pHBV1.2 plasmids, as indicated; after 48 h, the cells and supernatant were harvested and subjected to qPCR or ELISA. Data are representative of at least three independent experiments. Mean (±SD) values from three independent experiments are shown. **, *P* < 0.01; ***, *P* < 0.001.

Next, to identify the region of CH25H that interacted with HBx, CH25H was divided into two truncations, which included the transmembrane domain and FA domain, respectively (NCBI accession no. NP_003947.1) (Fig. 6A). Interestingly, both of the truncations interacted with HBx (Fig. 6B) and blocked the nuclear translocation of HBx protein (Fig. 6C), which resulted in a similar inhibition of HBV replication (Fig. 6D). Importantly, it was found that the transmembrane domain of CH25H included three regions, one of which (number 3) crossed over the transdomain and FA domain (NCBI accession no. NP_003947.1) (Fig. 6A). Further, it was assessed whether this region was critical for HBx interaction and HBV inhibition. As expected, number 3 deletion showed much weaker interaction with HBx than number 1 and number 2 deletions, which indicated number 3 is responsible for CH25H–HBx interaction (Fig. 6E). The functional assay further confirmed the importance of number 3 in the inhibition of HBV replication (Fig. 6F). Altogether, these findings suggested that CH25H interacted with HBx through the number 3 transmembrane region, and this region was important for the inhibition of HBV replication.

### 25HC promoted HBV infection but not HBV replication.

**FIG 7:**
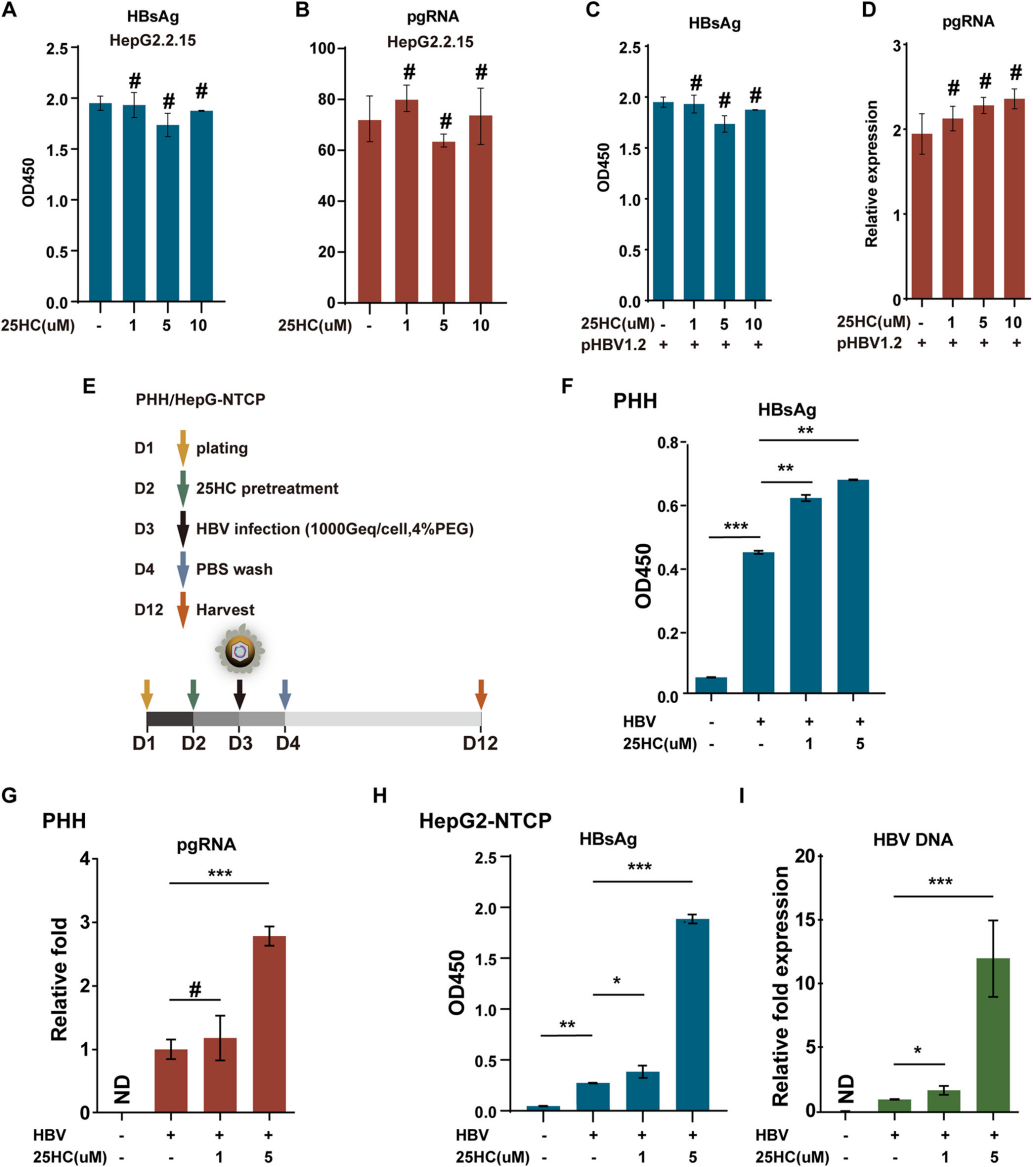
25HC promotes HBV infection. (A and B) HepG2.2.15 cells were treated with 25HC for 48 h, as indicated, and the cells and supernatant were harvested and subjected to qRT-PCR or ELISA to analyze the pgRNA or HBsAg level. (C and D) HepG2 cells were transfected with pHBV1.2 plasmids, and after 16 h, the cells were treated with 25HC, as indicated, for another 48 h, followed by cells and supernatant harvesting and analyses as described for panel A. (E) The scheme of HBV infection in PHH or HepG2-NTCP cells. (F to I) PHH (2.8 × 10^5^/well in a 24-well plate) or HepG2-NTCP cells (0.8 × 10^5^/well in a 12-well plate) were pretreated with 25HC in different doses (1 or 5 μM) for 24 h, followed by washing the cells with PBS and incubating for 24 h with HBV. After infection, the cells were washed with PBS and maintained for another 9 days; the medium was changed every 2 days. The supernatant and cells were collected for the detection of HBV DNA, pgRNA, or HBsAg by qRT-PCR or ELISA. Mean (±SD) values from three independent experiments are shown. *, *P < *0.05; **, *P < *0.01; ***, *P* < 0.001; #, *P* > 0.05.

To further verify the functions of 25HC in HBV replication, different doses of 25HC were added to treat the HepG2.2.15 cells. Interestingly, no inhibition of HBV replication was observed after the treatment (Fig. 7A and B). This result was further confirmed in pHBV1.2-transfected cells (Fig. 7C and D and Fig. S3A). In addition, following treatment with the supernatant obtained from CH25H WT- or Mut-transfected 293T cells, no obvious differences were noted for the HBsAg levels in the supernatant of pHBV1.2-transfected HepG2 cells, which further indicated a negative role of 25HC in the regulation of HBV replication (Fig. S3B). Moreover, 25HC was reported to inhibit viral infections by blocking the cell–virus membrane fusion. Thus, it was assessed whether 25HC played a role in the HBV infection process. For this, primary hepatocytes (PHH) and HepG2‐NTCP cells were used for HBV infection. In particular, cells were pretreated with 25HC for 24 h with different doses and infected with HBV for 24 h (Fig. 7E). After 8 days of infection, HBV DNA and HBsAg were measured in the supernatants by qPCR or enzyme-linked immunosorbent assay (ELISA), and pgRNA in the PHH cells was analyzed by qPCR. The results indicated that HBsAg, HBV DNA, or pgRNA were significantly upregulated by the 25HC treatment in a dose-dependent manner in both PHH (Fig. 7F and G) and HepG2-NTCP cells (Fig. 7H and I). As a positive control, an experiment was added to confirm the effort of 25HC in the inhibition of EV71 infection, and the same conditions were set. Interestingly, 25HC clearly inhibited EV71 infection and replication in both HepG2 and 293T cells (Fig. S4). Cell viability (of HepG2-NTCP, PHH, HepG2, and HepG2.2.15 cells) after 25HC treatment was qualified (Fig. S5). Altogether, our results indicated that 25HC did not affect the regulation of HBV replication but played an important role in the promotion of HBV infection.

10.1128/mbio.00677-22.3 FIG S3 25HC plays a negative role in HBV replication. (A) HepG2 cells were transfected with the pHBV1.2 plasmids for 16 h, after which the cells were treated with 25HC, as indicated, for another 48 h. The cells then were harvested and lysed for immunoblotting assay with anti-HBx, anti-HBs, or anti-GAPDH antibodies, as indicated. (B) Supernatants from empty vector, CH25H WT, or Mut transfected 293T cells were collected and added (0 to 1 mL) to HepG2 cells transfected with pHBV1.2 plasmids, as indicated; after 24 h of the treatment, HBsAg in the supernatant was detected by ELISA. Mean (±SD) values from three independent experiments are shown. #, *P > *0.05. Download FIG S3, TIF file, 1.0 MB. Copyright © 2022 Wei et al. 2022 Wei et al. https://creativecommons.org/licenses/by/4.0/ This content is distributed under the terms of the Creative Commons Attribution 4.0 International license.

10.1128/mbio.00677-22.4 FIG S4 25HC plays a negative role in HBV replication. (A) The scheme of EV71 infection in HepG2 or 293T cells. (B and C) HepG2 or 293T cells were pretreated with 25HC in different doses (1 or 5 μM) for 24 h, after which the cells were washed with PBS and incubated with EV71 for 36 h; the cells were finally washed with PBS and collected for the detection of EV71 expression by qPCR. **, *P* < 0.01; ***, *P* < 0.001. Download FIG S4, TIF file, 0.5 MB. Copyright © 2022 Wei et al. 2022 Wei et al. https://creativecommons.org/licenses/by/4.0/ This content is distributed under the terms of the Creative Commons Attribution 4.0 International license.

10.1128/mbio.00677-22.5 FIG S5 Cell viability assay. HepG2-NTCP, PHH, HepG2, or HepG2.2.15 cells were treated with 25HC, as indicated, and the cell viability was analyzed. Mean (±SD) values from 3 independent experiments are shown. *, *P* < 0.05; **, *P* < 0.01; #, *P* > 0.05. Download FIG S5, TIF file, 1.1 MB. Copyright © 2022 Wei et al. 2022 Wei et al. https://creativecommons.org/licenses/by/4.0/ This content is distributed under the terms of the Creative Commons Attribution 4.0 International license.

## DISCUSSION

Viral entry is the first step in the life cycle of the virus. In fact, it is a critical event that governs viral emergence into the host target cells (30). CH25H and its product 25HC are believed to play an antiviral role in response to viral infection by blocking the membrane fusion of the host cells and virus (3). The present study aimed to investigate the role of CH25H and 25HC in regulating HBV replication and infection. We found that CH25H interacted with HBx and inhibited its nuclear translocation, which resulted in the inhibition of HBV replication. Importantly, CH25H was found to bind to the C terminus of HBx, and transmembrane number 3 was verified to be indispensable for HBx interaction and HBV inhibition. 25HC was found to promote HBV infection in both HepG2-NTCP and PHH cells. The present study demonstrated a dual mechanism by which CH25H regulated HBV replication. The study provided evidence for cross talk between HBV and the host through CH25H–HBx axis.

HBx is a small protein that is encoded by the HBV genome. It is known to act as a transcription factor. HBx is indispensable for the life cycle of HBV, and it acts by promoting viral infection and replication (25). An inhibitor of NQO1 inhibits HBx expression by ubiquitin-independent degradation, which provided new insights into the regulation of HBx by chemical compounds (31, 32). HBx was shown to interact with DDB1, which was initially discovered as a DNA repair factor. This interaction assisted the integration of HBx into multisubunit complexes containing cullin-4A and other proteins, which further displayed E3 ligase activity (33). Smc5/6, a complex that is known to maintain chromosome stability, was degraded by the action of HBx–DDB1–CUL4 E3 ligase (19, 34). Therefore, the present study aimed to investigate the role of CH25H in this process. We found that CH25H is bound to the C terminus of HBx. This interaction did not inhibit the formation of the HBx–DDB1–Smc complex. However, CH25H–HBx interaction clearly inhibited the nuclear translocation of HBx, which further repressed HBV replication. HBx is a protein that is devoid of nuclear localization sequence (NLS). Interestingly, IκBα has been previously reported to be a partner of HBx. It plays a role in the promotion of HBx nuclear transport via direct interaction (26). In concordance with the results of the present study, it was previously reported that CH25H protein majorly exists in the cytoplasm, particularly in endoplasmic reticulum (ER) (35). Therefore, it could be suggested that the interaction of CH25H blocks HBx transport from the cytoplasm to the nucleus; however, more study needs to be done to further explore the detailed mechanism of HBx transport.

In response to HBV infection, the induction of interferon has been shown to be inconsistent. It was reported that interferon signaling was not activated in the liver samples of chronic HBV infection patients (36). HDV but not HBV infection strongly activated IFN-β and a broad range of interferon-stimulated genes (ISGs) in HepG2-NTCP cells (37); however, IFN-α, IFN-β, IFN-λ, interleukin-29 (IL-29), and IL-6 were found to be induced within 24 h after HBV infection in HepaRG cells (38), and in another study, type III but not type I or II interferon was upregulated after HBV infection (39). In our previous study, we found that CBFβ was induced by type III interferon at the early stage of HBV infection (16). In conformance, CH25H, as an interferon-stimulated gene (3), was found to be upregulated in the HBV patients’ peripheral blood mononuclear cells (PBMCs) compared to those of the healthy controls in the present study. However, high CH25H expression was found to be correlated with low HBV DNA copy numbers and HBsAg levels in the patients. As is already known, PBMC is not the target cell of HBV; therefore, HBsAg- or HBV-related products in the blood are assumed to regulate the expression of CH25H (10). On the other hand, cytokines like type III interferon or IL-6, which were induced in response to HBV infection, may also increase the CH25H expression in both hepatocytes and PBMCs (39, 40), and the high expression of CH25H in the hepatocytes may inhibit HBV replication. Thus, the expression of CH25H reflects the balance of the host and virus interaction, and CH25H may be different at different stages of the HBV infection.

25HC was previously reported to activate CH25H expression in an LXR-dependent manner (35). Liver X receptors (LXRs), including LXRα (NR1H3) and LXRβ (NR1H2), are known to play various biological roles, especially in lipid metabolism (41). Among these, LXRα is abundantly expressed in the liver tissue and some other tissues, like adipose tissue. In comparison, LXRβ expression can be detected in the majority of tissues. Interestingly, it was previously shown that treatment with LXR agonists could elicit potent anti-HBV activity in PHHs and dHepaRG cells, possibly through sustained suppression of cccDNA transcription. However, similar inhibition was not observed in the case of HepG2-derived hepatoma cell lines, such as HepG2.2.15, HepDE19/HepDES19, and HepG2/NTCP cell lines (42). However, another study showed that the addition of oxysterols, such as 22(R)‐HC and T0901317, to HepG2 cells transfected with HBV genome-containing plasmid resulted in increased HBV gene expression and viral promoter activity, which was directed by induction of nuclear receptor LXR. Interestingly, when IFN-α and oxysterols were coincubated, oxysterols and LXR significantly reduced the anti-HBV effects of IFN (9). LXR activation was also previously shown to enhance CVB3 viral replication (43). Interestingly, HBx was reported to interact with LXR-α and enhanced LXRE binding and expression of target genes (44). The present study assessed whether CH25H interacted with HBx and inhibited HBx nuclear translocation, which further inhibited HBV replication. As the product of CH25H, 25HC is known to play a promotional role in HBV replication. The treatment with 25HC might enhance the expression of some LXR downstream genes, which could benefit the infection of HBV. Nevertheless, further investigation is required to explore the detailed mechanisms.

**FIG 8:**
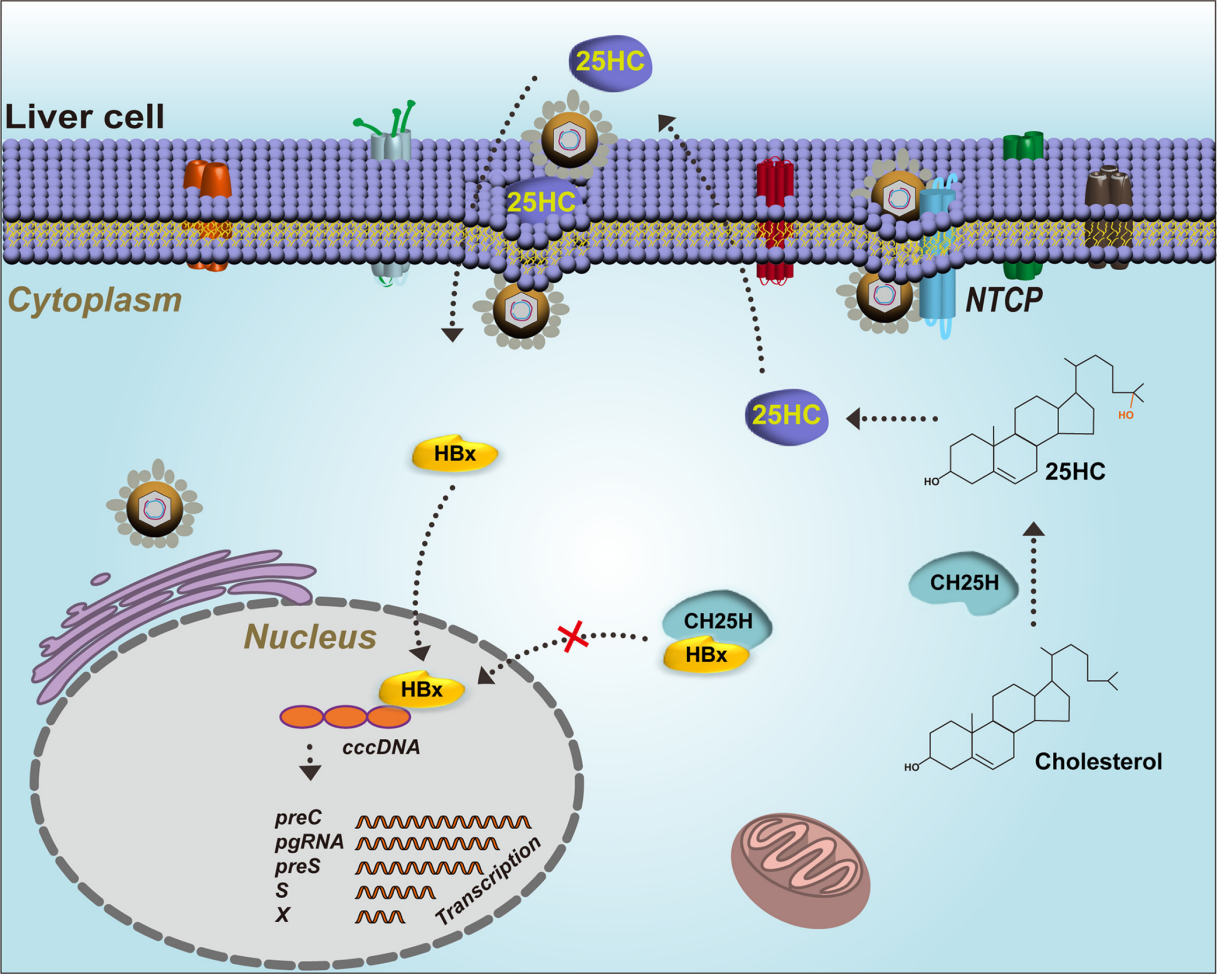
Model depicting the dual role of CH25H in regulating the replication of hepatitis B virus. Briefly, on one hand, CH25H interacts with HBx protein and inhibits HBx nuclear translocation, which reduces HBV transcription from the cccDNA template. On the other hand, CH25H promotes the production of 25HC by catalyzing cholesterol hydroxylation, and the secreted 25HC promotes the infection of HBV.

On the basis of all these findings, the present study proposed a working model for CH25H-mediated dual regulation of HBV replication and infection (Fig. 8). In conclusion, it was observed that CH25H was upregulated in HBV patients. Importantly, higher expression of CH25H in the patients correlated with low HBV DNA copy numbers, which indicated a positive role of CH25H in the regulation of HBV replication. In terms of mechanism, transmembrane region number 3 of CH25H was found to interact with the C terminus of HBx protein, which blocked the nuclear translocation of HBx protein and inhibited HBV replication. On the other hand, 25HC, the enzyme-catalyzed product of CH25H, promoted the infection of HBV in the HBV infection model and played a negative role in HBV replication. Thus, the present study suggested a new mechanism for the role of CH25H in the regulation of viral infection and replication, which provided a detailed understanding of the cross talk between the host and virus. The findings of the study might further provide a foundation for the research and development of small-molecule drugs.

## MATERIALS AND METHODS

### Samples.

Forty-eight HBV-infected patients and 22 healthy controls were enrolled in this study (see Table S1 in the supplemental material). Venous blood samples (5 mL) were collected to obtain the serum and PBMCs. The HBV DNA level was detected using Roche’s COBAS TaqMan kit. The liver function and the biochemical parameters were assayed by using an automatic biochemical instrument. The tests were conducted at the Hepatology Department of the First Hospital of Jilin University, Changchun, China. The study protocol was approved by the IRB of Jilin University, the First Hospital.

10.1128/mbio.00677-22.6 TABLE S1 Characteristics of the study population. Download Table S1, DOCX file, 0.02 MB. Copyright © 2022 Wei et al. 2022 Wei et al. https://creativecommons.org/licenses/by/4.0/ This content is distributed under the terms of the Creative Commons Attribution 4.0 International license.

### Cell culture, plasmids, and reagents.

Human embryonic kidney 293T (HEK293T), HepG2, HepG2.2.15, and HepG2-NTCP cells were maintained in Dulbecco’s modified Eagle medium (DMEM) containing 10% inactivated fetal bovine serum, penicillin (100 IU/mL), and streptomycin (100 mg/mL) and incubated at 37°C under 5% CO_2_ atmosphere. Primary hepatocytes (PHH; M00995-P; RILD, Shanghai, China) were plated with plating medium containing 10% fetal bovine serum (FBS) (S03316; RILD, Shanghai, China) and maintained with incubation medium (Z99009; RILD, Shanghai, China). The expression construct of CH25H, HBx, and truncations was generated by cloning the coding region sequence of the relative human genes into the VR1012 vector with Flag, hemagglutinin (HA), or glutathione-*S*-transferase (GST) tag according to the instructions of the EASY-Uni seamless cloning kit (TransGen, Beijing, China). Site-directed mutagenesis of CH25H was generated by QuikChange PCR (TransGen). The primers used in this study are listed in Table S2.

10.1128/mbio.00677-22.7 TABLE S2 Primers for infusion cloning. Download Table S2, DOCX file, 0.02 MB. Copyright © 2022 Wei et al. 2022 Wei et al. https://creativecommons.org/licenses/by/4.0/ This content is distributed under the terms of the Creative Commons Attribution 4.0 International license.

### Total RNA and HBV DNA extraction and quantitative real-time PCR.

Total RNA was extracted with TRIzol (Invitrogen, San Diego, CA, USA) and then converted to first-strand cDNA using Superscript III transcriptase (Invitrogen). HBV DNA was isolated from the whole-cell lysate or culture supernatant according to the manufacturer's instructions (TransGen, Beijing, China). Glyceraldehyde-3-phosphate dehydrogenase (GAPDH) was used as an internal control; quantitative real-time PCR was performed as described elsewhere (45). The sequences of gene-specific primer used for qPCR are given in Table S3.

10.1128/mbio.00677-22.8 TABLE S3 Primers sequences for quantitative real-time PCR. Download Table S3, DOCX file, 0.02 MB. Copyright © 2022 Wei et al. 2022 Wei et al. https://creativecommons.org/licenses/by/4.0/ This content is distributed under the terms of the Creative Commons Attribution 4.0 International license.

### Coimmunoprecipitation and Western blotting.

The experimental cells were lysed 24 to 48 h after transfection with the expression plasmids using 50 mM Tris-HCl (pH 8.0), 150 mM NaCl, and 1% NP-40 containing cocktail inhibitors (Roche, USA). For immunoprecipitation, the lysates were incubated overnight with the anti-FLAG M2 affinity gel (Sigma, USA). Immunoblotting was carried out as described elsewhere (46). Briefly, the cells were collected and lysed by adding radioimmunoprecipitation assay (RIPA) lysis buffer together with protease/phosphatase inhibitor cocktail on an ice bath for 30 min and by tapping tubes every 10 min. The protein concentration was quantified by the Coomassie Plus protein assay reagent (Thermo Scientific, Rockford, IL, USA). The quantification of immunoblotting band intensity was performed with the ChemiDoc XRS^+^ Molecular Imager software (Bio-Rad, Philadelphia, PA, USA). The samples were separated by SDS-PAGE and transferred onto polyvinylidene difluoride (PVDF) membranes. After blocking in Tris-buffered saline (TBS) containing 0.1% Tween 20 and 5% skim milk, the blots were probed with relative antibodies.

### Nuclear and cytoplasmic extraction.

To prepare the nuclear and cytoplasmic fractions, the cells were treated as indicated by the nuclear and cytoplasmic protein extraction kit (Beyotime, China). The purified cytoplasmic and nuclear fractions were subjected to Western blotting assay according to the standard procedures with the relevant antibodies.

### Immunofluorescence.

HepG2 cells were transfected with Flag-HBx plasmids or together with HA-CH25H plasmids for 48 h, and the cells were then fixed in acetone-methanol (1:1) at 37°C for 10 min. Subsequently, the cells were washed with phosphate-buffered saline (PBS) and blocked with 5% bovine serum albumin (BSA) in PBS-Tween 20 (PBST) for 1 h, followed by incubation with Flag (mouse) and HA (rabbit) antibody at 37°C for 1 h, washing in PBS, and incubating with goat anti-mouse IgG conjugated with fluorescein isothiocyanate (FITC)- or both Cy3 (rabbit)- and FITC (mouse)-conjugated IgG (Proteintech). The cells were washed with PBS and observed by fluorescence microscopy.

### ELISA.

HepG2 cells were mock transfected or transfected with CH25H expression plasmids together with the pHBV1.2-HBV expression plasmids. The supernatant was collected after 72 h, and the supernatant from HBV-infected HepG2-NTCP cells was collected after 9 days for analysis by ELISA to detect the HBsAg levels (Kehua, Shengwu, China).

### HBV infection assay.

The serum from HBV patients infected with different virus strains (HBV DNA copies, >10^7^) was collected, and the whole virus was concentrated using PEG-it virus precipitation solution (System Biosciences, USA). Lentivirus expressing CH25H WT or Mut were purchased from Genecopoeia (numbers LPP-NEG-Lv201-100, LPP-Q0427-Lv201-100, and LPP-CS-Q0427-Lv201-01-100), and the HepG2-NTCP stable cell line was obtained by infecting cells with the lentivirus and then selected with puromycin. HepG2-NTCP or PHH cells were pretreated with 25HC for 24 h and then inoculated with the serum-produced virus at a multiplicity of infection (MOI) of 1,000 equivalents (Geq) per cell; the cells were then cultured in the presence of 4% polyethylene glycol 8000 and 2% dimethyl sulfoxide (DMSO) for 24 h. After infection, the cells were washed thrice with PBS and maintained in the DMEM (HepG2-NTCP) or incubation medium (PHH) for another 9 days; the medium was changed every 2 days. The supernatant and cells were collected for the detection of HBV DNA, pgRNA, and HBsAg by qPCR or ELISA.

### Quantification and statistical analyses.

GraphPad Prism 8 software (GraphPad Software, San Diego, CA) was used for data analyses; a two-tail unpaired *t* test was used to assess between-group differences. A *P *value of <0.05 was considered to indicate statistical significance.

### Data and code availability.

The published article includes all data sets generated or analyzed during this study.
