# Supplementary figures and images for: Dual-Role of Cholesterol‐25‐Hydroxylase in Regulating Hepatitis B Virus Infection and Replication
Source: mBio. 2022 May 19;13(3):e00677-22. doi: 10.1128/mbio.00677-22 (PMC9239238; doi:10.1128/mbio.00677-22)

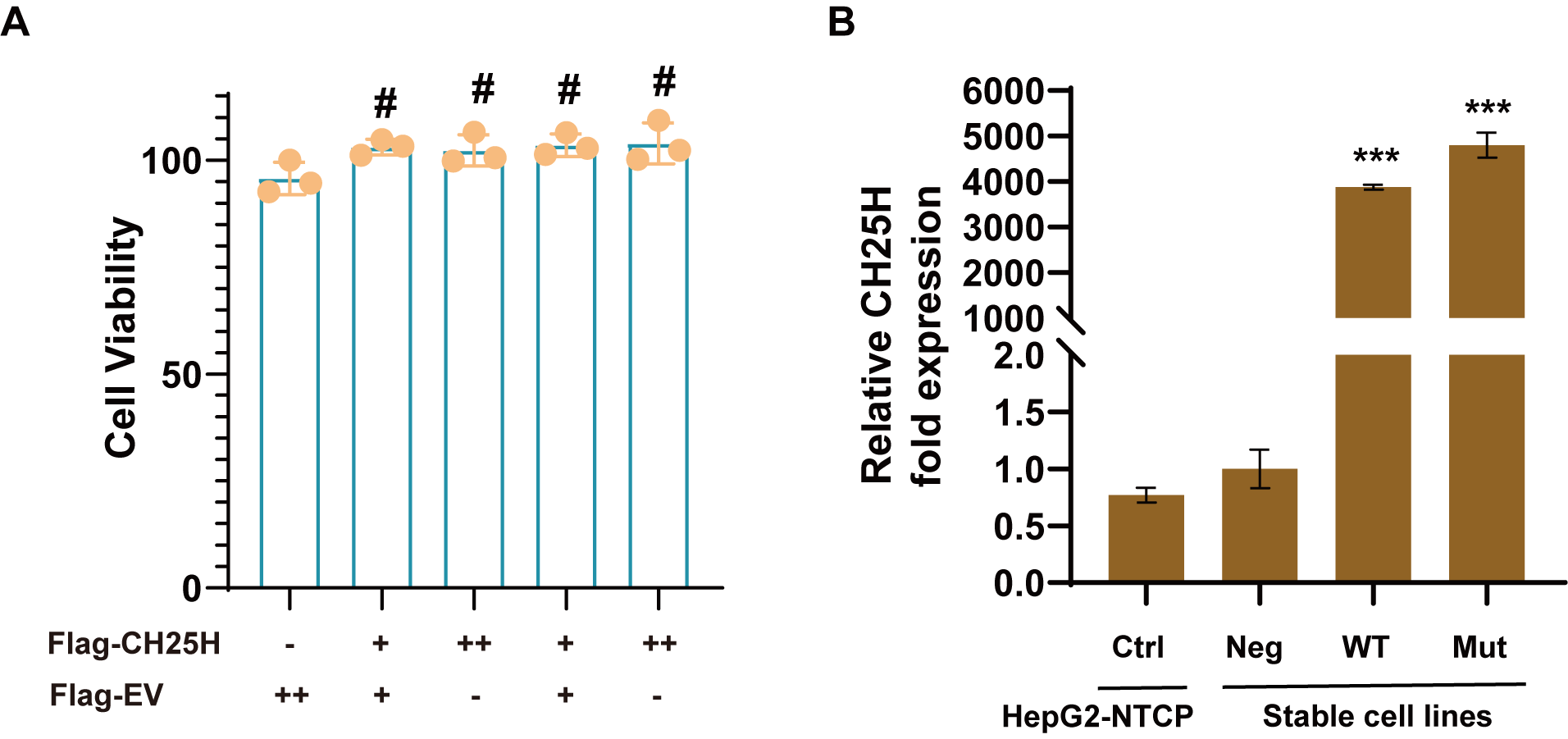

Supplement: FIG S1 [file mbio.00677-22-s0001.tif]

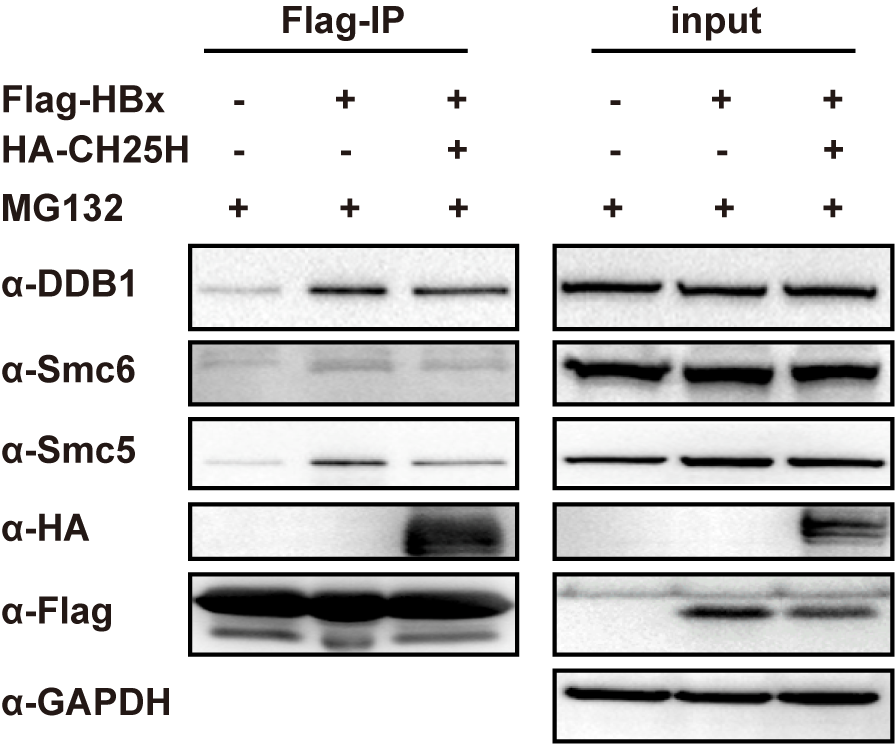

Supplement: FIG S2 [file mbio.00677-22-s0002.tif]

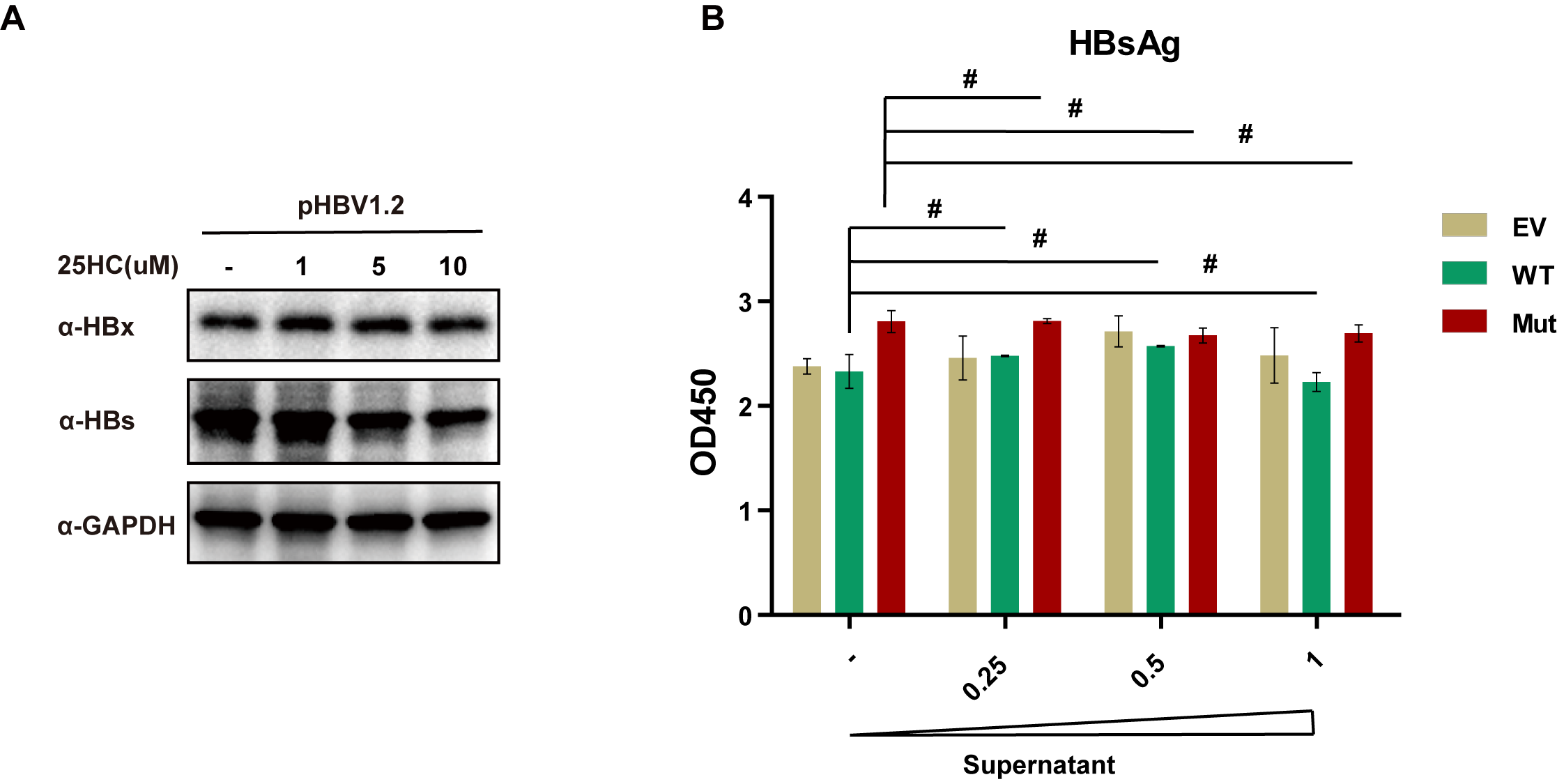

Supplement: FIG S3 [file mbio.00677-22-s0003.tif]

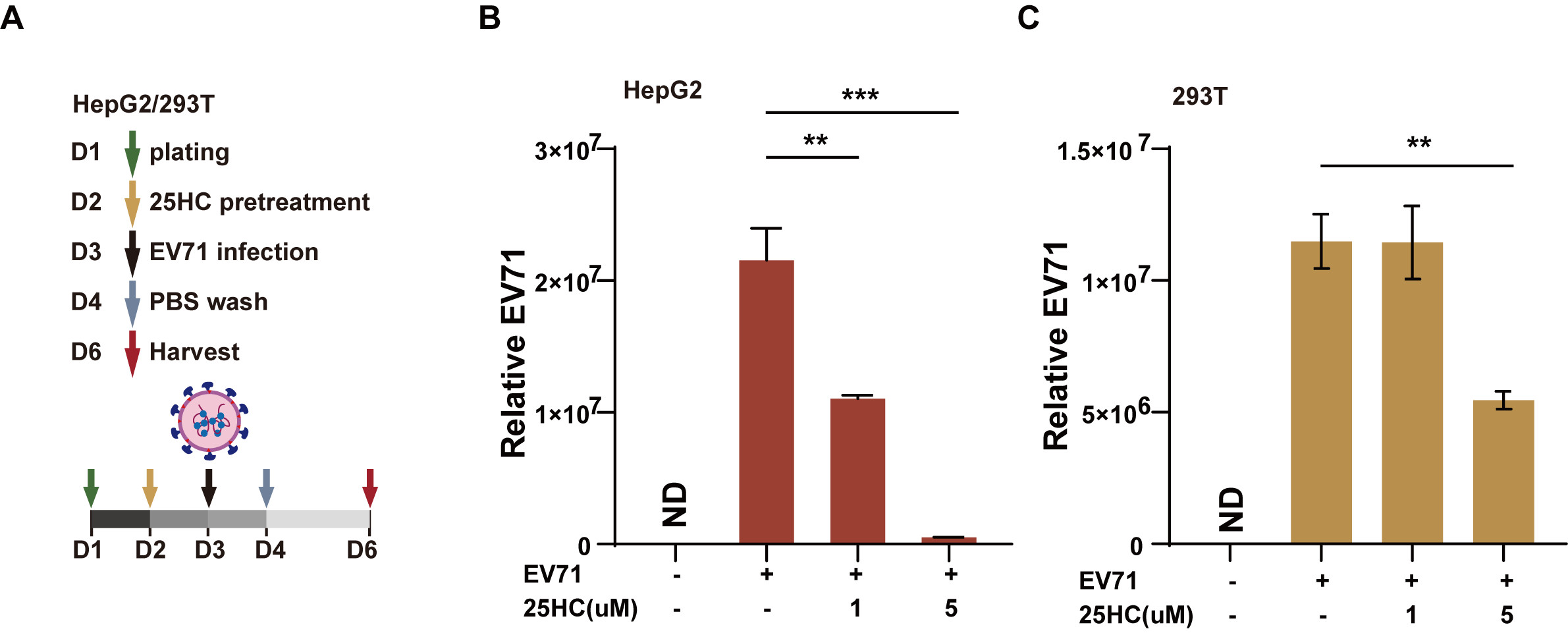

Supplement: FIG S4 [file mbio.00677-22-s0004.tif]

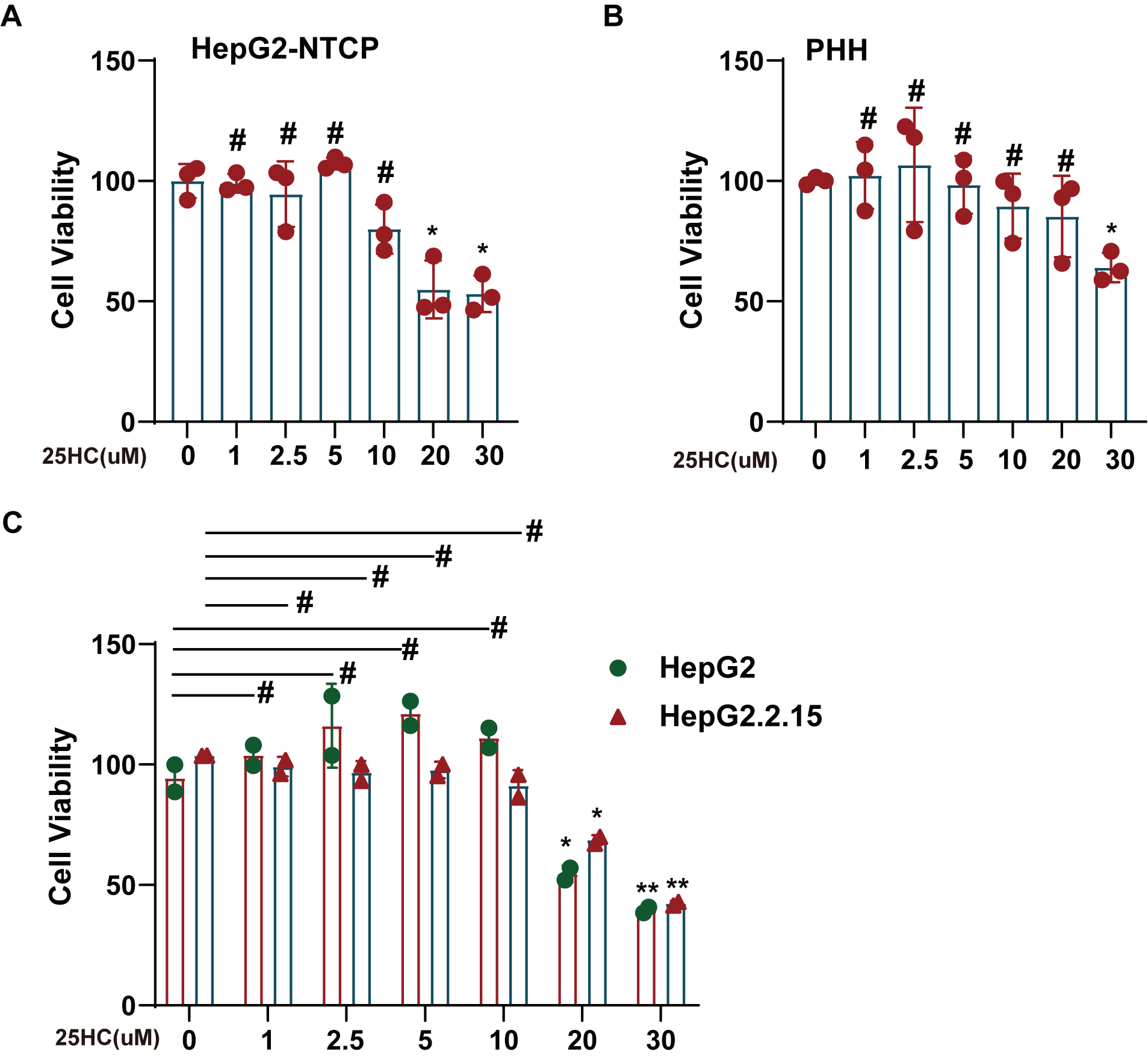

Supplement: FIG S5 [file mbio.00677-22-s0008.tif]
