# Supplementary material for: Dual-Role of Cholesterol‐25‐Hydroxylase in Regulating Hepatitis B Virus Infection and Replication
Source: mBio. 2022 May 19;13(3):e00677-22. doi: 10.1128/mbio.00677-22 (PMC9239238; doi:10.1128/mbio.00677-22)
Supplement: TABLE S1 [file mbio.00677-22-s0005.docx]

**Table S1. Characteristics of the study population**

| Clinical data | Healthy controls  (n = 22) | HBV patients  (n = 48) |
| --- | --- | --- |
| Sex (male/female) | 10/12 | 27/21 |
| Age (y) | 36.5 $\pm$13.61 | 44.75 $\pm$11.96 |
| ALT (U/L) | 20.01 $\pm$14.65 | 79.88 $\pm$94.60 |
| AST (U/L) | 21.77 $\pm$8.751 | 77.80 $\pm$109.9 |
| HBsAg (IU/ml) | Negative | 13958 $\pm$22586 |
| HBeAg (S/CO) | Negative | 254.1 $\pm$509.5 |
| HBcAb (S/CO) | Negative | 8.865 $\pm$3.088 |
| HBV DNA (log10 IU/ml) | Negative | 4.843 $\pm$2.148 |
| LDL (mmol/L) | 2.499 $\pm$0.5492 | 2.940 $\pm$0.9962 |
| AFP (ng/ml) | Negative | 34.29 $\pm$143.7 |

Data are presented as average values $\pm$SD.

S/CO, signal to cut-off ratio.
