# Supplementary material for: Dual-Role of Cholesterol‐25‐Hydroxylase in Regulating Hepatitis B Virus Infection and Replication
Source: mBio. 2022 May 19;13(3):e00677-22. doi: 10.1128/mbio.00677-22 (PMC9239238; doi:10.1128/mbio.00677-22)
Supplement: TABLE S2 [file mbio.00677-22-s0006.docx]

**Table S2: Primers for infusion cloning**

**CH25H**

| Primers | | Sequence |
| --- | --- | --- |
| WT | Forward | 5'-TCGCGGCCGCTCTAGAATGAGCTGCCACAACTGCTC-3' |
| WT | Reverse | 5'-AGGCGCCTGGTCTAGATCACCGCGCTGGGACAGATG-3' |
| TM | Forward | 5'-TCGCGGCCGCTCTAGAATGAGCTGCCACAACTGCTC-3' |
| TM | Reverse | 5'-AGGCGCCTGGTCTAGATTAGAAGAGTAGCAGGCAGAA-3' |
| FA | Forward | 5'-TCGCGGCCGCTCTAGAATGGACATGGAGTTCTTCGTG-3' |
| FA | Reverse | 5'-AGGCGCCTGGTCTAGATCACCGCGCTGGGACAGATG-3' |
| DEL 1 | Forward | 5'-CAGTCGCCCGATATCCTGTGCTCC-3’ |
| DEL 1 | Reverse | 5'-CAGGATATCGGGCGACTGTAGGAG-3’ |
| DEL 2 | Forward | 5'-GCGCAGCAGCTGCATTGGGCCCGC-3’ |
| DEL 2 | Reverse | 5'-CCAATGCAGCTGCTGCGCGGATGG-3’ |
| DEL 3 | Forward | 5'-GCTCCCGAGTGGCACCTGCTGCAC-3’ |
| DEL 3 | Reverse | 5'-CAGGTGCCACTCGGGAGCTTCGTG-3’ |

**HBx**

| Primers | | Sequence |
| --- | --- | --- |
| WT | Forward | 5'-TCGCGGCCGCTCTAGAATGGCTGCTCGGGTGTGCTGC-3' |
| WT | Reverse | 5'-AGGCGCCTGGTCTAGATTAGGCAGAGGTGAAAAAG-3' |
| X1 | Forward | 5'-TCGCGGCCGCTCTAGAATGGCTGCTCGGGTGTGCTGC-3' |
| X1 | Reverse | 5'-AGGCGCCTGGTCTAGATTACCCGTGGTCGGCCGGAAC-3' |
| X2 | Forward | 5'-TCGCGGCCGCTCTAGAATGGCGCACCTCTCTTTAC-3' |
| X2 | Reverse | 5'-AGGCGCCTGGTCTAGATTAGAGTCCAAGAGTCCTC-3' |
| X3 | Forward | 5'-TCGCGGCCGCTCTAGAATGTCAGCAATGTCAACGACC-3' |
| X3 | Reverse | 5'-AGGCGCCTGGTCTAGATTATTAGGCAGAGGTGAAAAA-3' |
| DEL | Forward | 5'-TCGCGGCCGCTCTAGAATGGCTGCTAGGCTGTGCTGC-3' |
| DEL | Reverse | 5'-AGGCGCCTGGTCTAGATTACCTATTCTCCTCCCC-3' |
