# Supplementary material for: Dual-Role of Cholesterol‐25‐Hydroxylase in Regulating Hepatitis B Virus Infection and Replication
Source: mBio. 2022 May 19;13(3):e00677-22. doi: 10.1128/mbio.00677-22 (PMC9239238; doi:10.1128/mbio.00677-22)
Supplement: TABLE S3 [file mbio.00677-22-s0007.docx]

**Table S3: Primers sequences for quantitative real-time PCR**

| Primers | | Sequence |
| --- | --- | --- |
| GAPDH | Forward | 5'-CGGATTTGGTCGTATTGGG-3' |
| GAPDH | Reverse | 5'-TCTCGCTCCTGGAAGATGG-3' |
| CH25H | Forward | 5′-CTTTCCGTGGAGGACCACTC-3′ |
| CH25H | Reverse | 5′-TACGGAGCGAAGTTGCAGTT-3′ |
| HBV DNA | Forward | 5'-GAGTGTGGATTCGCACTCC-3' |
| HBV DNA | Reverse | 5'-GAGGCGAGGGAGTTCTTCT-3' |
| HBV pgRNA | Forward | 5'-TCTTGCCTTACTTTTGGAAG-3' |
| HBV pgRNA | Reverse | 5'-AGTTCTTCTTCTAGGGGACC-3' |
| HBx | Forward | 5'-ACGTCCTTTGTTTACGTCCCGT-3’ |
| HBx | Reverse | 5'-CCCAACTCCTCCCAGTCCTTAA-3’ |
| CVB3 | Forward | 5'-ATGAGACCAGGCTGAATGC-3’ |
| CVB3 | Reverse | 5'-TACTGGTTCTGTGAACTTGC-3’ |
| EV71-VP1 | Forward | 5'-AGCACCCACAGGCCAGAACACAC-3’ |
| EV71-VP1 | Reverse | 5'-ATCCCGCCCTACTGAAGAAACTA-3’ |
